# Depression and Resilience in Breast Cancer Patients

**DOI:** 10.3889/oamjms.2015.119

**Published:** 2015-11-13

**Authors:** Gordana Ristevska-Dimitrovska, Petar Stefanovski, Snezhana Smichkoska, Marija Raleva, Beti Dejanova

**Affiliations:** 1 *University St. Kliment Ohridski Bitola, Higher Medical School Bitola, Bitola, Republic of Macedonia*; 2 *Clinical Hospital Dr. Trifun Panovski, Department of Oncology, Bitola, Republic of Macedonia*; 3 *University Clinic for Radiotherapy and Oncology, Medical Faculty, Ss Cyril and Methodius University of Skopje, Skopje, Republic of Macedonia*; 4 *University Clinic of Psychiatry, Faculty of Medicine, Ss Cyril and Methodius University of Skopje, Skopje, Republic of Macedonia*; 5 *Department of Medical and Experimental Physiology with Anthropology, Medical Faculty, Ss Cyril and Methodius University of Skopje, Skopje, Republic of Macedonia*

**Keywords:** Connor Davidson Resilience Scale, Hospital Anxiety Depression Scale, depression, resilience, breast cancer

## Abstract

**OBJECTIVE:**

A significant number of breast cancer patients, during their life with the diagnosis, experience emotional distress in the form of depression and anxiety. Psychological resilience is the ability of a person to protect his/her mental health when faced with adverse circumstances such as the cancer diagnosis. This study aims to assess the resilience in breast cancer patients and to explore whether depression affects the resilience.

**MATERIAL AND METHODS:**

Two hundred eighteen (218) women, treated for early breast cancer responded to Connor - Davidson Resilience Scale and Hospital Depression and Anxiety Scale, in order to assess the level of psychological resilience and the level of depression.

**RESULTS:**

There is a significant negative correlation between depression and resilience in our sample (r = - 0.562, p < 0.001). Individuals with higher levels of depression have lower levels of psychological resilience. There is no statistically significant correlation between the ages of the participants; time passed since diagnosis, cancer stage and resilience levels.

**CONCLUSION:**

This study shows that patients who are less depressed have higher levels of resilience and that psychological resilience may independently contribute to lower levels of depression among breast cancer patients. The level of psychological resilience may be a protective factor for depression and psychological distress.

## Introduction

Psychosocial and behavioral studies of cancer survivors show a wide variety of effects of the disease and treatments upon the lives of patients and their families. These studies come to the conclusion that being a “disease-free” does not mean to be “free from the disease.” Cancer has power to influence all aspects of the human health: physical, functional, psychological/cognitive, social, economic and spiritual. Through conversation and following the life stories of people being treated for cancer, researchers have noted that although the experience is often catastrophic, many patients show high level of resilience when faced with disease.

Cancer patients suffer from much different psychiatric comorbidity, but the most frequently diagnosed are depressive disorder, anxiety disorders (panic disorder, post-traumatic stress disorder, and phobias), adjustment disorders and delirium [1]. Often, patients have mixed condition or a combination of symptoms such as depression and anxiety.

In the literature, one can find many different data on the prevalence of clinically significant depression in oncology patients. These differences depend on the location and stage of cancer, the demographic characteristics of examined population, diagnostic criteria and instruments used, method of assessment, and many other factors [2]. Generally, it is estimated that 10% to 25% of cancer patients have clinically significant depressive symptoms [3, 4], with higher prevalence in late life of patients [5], and in malignant diseases with worse prognosis, such as pancreatic cancer and oropharyngeal cancer [6, 7]. It is believed that the incidence of depression in patients with cancer is 3 to 4 times more common than in the general population [8]. Potential reasons for the inconsistency in the literature include differences in methodology (many different tools for screening and diagnosis of depression are used), there are too much differences in defining the research population, and often the entire population of patients with breast cancer is comprised without taking into consideration the singularity of time period passed since the diagnosis [9].

Depression in patients with malignant disease contributes to reduced quality of life [10], a higher rate of suicide and desire for hastened death [11], poorer adherence to the oncology treatment [12], greater physical suffering [13] and prolonged hospital stays [14]. There is evidence that depression is an independent risk factor for mortality in patients with malignant diseases [15-18].

Cancer as a diagnosis is associated with psychological distress across all points of the trajectory of the disease. Many factors contribute to this psychological suffering such as the feeling of uncertainty about the future, loss of the autonomy and the sense of control over the changes in life and the possibility of disability, fear of suffering and death and existential worries [19].

Patients with breast cancer show a higher rate of five-year survival compared to other malignancies in women. It is believed that the survival rate of patients with breast cancer is between 80% and 95% [20]. The diagnosis of breast cancer is no longer considered as a fatal diagnosis, and is more and more accepted as a disease with positive treatment outcomes. Although the disease is successfully treated, the actual diagnosis of cancer and treatment that patients must undergo are very stressful. Clinicians should be aware of the frequency of depression in cancer patients and early identify the psychological symptoms. Early detection and treatment of depression in patients with breast cancer improves the patients’ quality of life and increases the probability of successful recovery [21]. The period after completion of the initial oncologic treatment is also a time of transition when women need to transform their role of “breast cancer patient” to the role of “symptom-free patient” or “survivor” [22]. The depressive disorder is often underestimated in patients with breast cancer [23, 24]. It is considered that depressive disorder is most common in the period when the diagnosis of breast cancer is established. The depressive disorder later in life after the initial therapy for breast cancer is not thoroughly researched. During regular oncological follow-up examinations, psychological needs of patients living with no signs of cancer are often overlooked [25]. One of the prominent causes for development of depression among breast cancer survivors is the fear of cancer recurrence or occurrence of metastases [26].

Very important psycho-social factor regarding the psychological suffering of cancer patients is their resilience. Resilience implies the ability of a person to protect or recover his/her mental health despite the existence of objective difficulties. Resilience is not a single feature of the person but it is a result of the interactions between multiple personality traits and environmental factors [27]. It is believed that higher levels of resilience protect a person from psychiatric disorders. In cancer patients, higher levels of resilience are associated with better quality of life and lower levels of depression in patients [28]. Given that most resilience studies in cancer patients are cross-sectional studies, it is difficult to derive a reliable conclusion whether people who are resilient suffer less from psychological distress or the lesser psychological suffering makes people more resilient when dealing with an adversity. The ability of patients to protect their mental health and experience positive psychological growth when faced with catastrophic experience is insufficiently explored.

This study aims to assess the resilience in breast cancer patients and to explore whether depression affects the resilience of patients suffering from breast cancer.

## Material and Methodology

The study was conducted in the oncology department of the Clinical Hospital “Dr. Trifun Panovski “ in Bitola and University Clinic for Oncology and Radiotherapy in Skopje. Participation in the study was offered to all patients who came for a follow-up in the oncology wards in Bitola and Skopje and who met the inclusion criteria. Data for 218 patients have been compiled and analyzed in this study, women treated for breast cancer, older than 18 years, treated with different oncological therapies and that are in a different time period from the initial treatment of malignant disease.

### Inclusion criteria

The diagnosis of early breast cancer to be based on histopathology analysis (stage I, II and III); Patients with breast cancer can be included in the study if they have completed the initial surgical and oncological treatment at least 1 month prior the interview; Patients who have or have had metastasis cannot be included in the study; Patients who have comorbid or pre-existing brain disorders in the form of delirium, encephalopathy or other disorder in the course of treatment were not included in the study; Patients included in the study had no history of psychiatric illnesses (schizophrenia spectrum, schizotypal and delusional disorders, affective disorders, diagnosed personality disorders, addiction) before the diagnosis of breast cancer.

**Figure 1:**
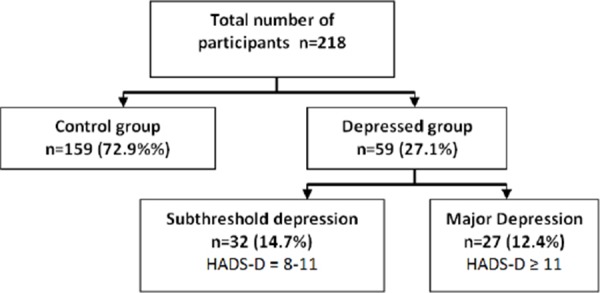
*Depression according to HADS scale, cut-off HADS-D ≥ 8*.

Participants answered the following questionnaires:

1. Questionnaire for psychosocial and oncologic parameters specifically designed for the study.
2. Connor-Davidson Resilience Scale 25 (CD-RISC25) [29, 30], is a self-evaluation instrument of 25 items that are ranked on a Likert scale from 0 to 4 points. The full range of the scale is from 0-100 points, where higher scores indicate higher resilience.
3. Hospital Anxiety Depression Scale (HADS) [31-33], - for self-assessment of anxiety and depression. The HADS scale contains 14 items: 7 for assessment of anxiety symptoms and 7 for assessment of depressive symptoms. For each item there are four possible answers scored from 0 to 3 points. The total sum of both subscales (depression, anxiety) is interpreted as follows: 0-7 no symptoms, 8-10 borderline case, 11 points and more than 11 indicates clinically significant anxiety or depression. In studies with cancer patients, researchers accepted to use cut-off ≥ 8 points on HADS-D subscale for clinically significant depression.

All participants have given a written informed consent for participation in the study. The study was approved by The Ethics Committee for research on people at the Medical Faculty in Skopje.

## Results

The mean resilience score of the whole group is 74.7 (0-100) SD 17.0, and the mean depression score on HADS-D is 5 (SD 4.2). The not depressed subgroup had mean resilience of 79.1 (SD 14.6), the subthreshold group had 68.2 (SD 14.3) and the major depression group had mean levels of resilience of 56.4 (SD 18.7).

**Table 1:** Summary of demographic and oncological data

Demographic Information		Cancer Diagnosis and Treatment	
Age / Range	30-90	**Stage**	**186**

Age / Mean (years)	60.2	I	52 (27.9%)

**Marital Status**	**216**	II	74 (39.8%)

Married	147 (68%)	III	60 (32.3%)

Never Married	13 (6%)	**Time from diagnosis**	**211**

Other (Divorced, widowed)	56 (25.9%)	6 months-1 year	23 (10.9%)

**People in household**	**217**	1-2 years	28 (13.2%)

Only the patient	27 (12.4%)	2-5 years	74 (35.1%)

Shares with other/s family members	190 (87.6%)	>5 years	86 (40.8%)

**Patient is parent**	**218**	**Type of Treatment**	**218**

Yes	198 (90.8%)	ChemoTh+RadioTh+Surgery	104 (47.7%)

No	20 (9.2%)	ChemoTh+Surgery	72 (33%)

**Age of Patients’ Children**	**192**	RadioTh+Surgery	11 (5%)

<10 years old	6	Surgery	31 (14.2%)

11-20	17 (8.9%)	**Type of Surgery**	**212**

21-30	55 (28.6%)	Partial Mastectomy	40 (18.9%)

>31	114 (59.4%)	Mastectomy	165 (77.8%)

**Education**	**217**	Double Mastectomy	7 (3.3%)

Primary School	56 (25.8%)	**Adjuvant Therapy (present)**	**206**

Secondary School	88 (40.6%)	Yes	134 (65%)

University	73 (33.6%)	No	72 (35%)

**Employment**	**214**	**Type of Medication**	**121**

Employed	58 (27.1%)	Tamoxifen	108 (89.2%)

Unemployed	50 (23.4%)	Aromatase Inhibitor	13 (10.8%)

Retired	106 (49.5%)	**Who financially supports the family?**	**214**

**Place of residence**	**200**	Patient alone	58 (27.1%)

Urban area	167 (83.5%)	Patient’s partner	36 (16.4%)

Rural area	33 (16.5%)	Patient and partner together	86 (40.2%)

**Family Net Monthly Income**	**214**	Other	34 (15.9%)

Up to 10000 MKD (160 EUR)	58 (27.1%)	**No of individuals in the social support network**	**195**

10000-30000 MKD (160-500 EUR)	116 (54.2%)	0 individuals	5 (2.6%)

>30000 MKD (>500 EUR)	40 (18.7%)	1-5 individuals	73 (37.4%)

		>5 individuals	117 (60%)

There is strong negative correlation between the level of resilience and the level of depression on the HADS-D subscale (r = - 0.562, p < 0.001). The level of resilience does not correlate with the age of participants (r = - 0.077; p = 0.263).

**Table 2:** Comparison of means of resilience in 3 groups of patients (not depressed, subthreshold depression, major depression)

Not depressed vs. Subthreshold Depression	t = 3.863, df =186	P < 0.001

Not depressed vs. Major Depression	t = 6.026, df =182	P < 0.001

Subthreshold Depression vs. Major Depression	t = 2.681, df =56	P = 0.01

## Discussion

The aim of the study was to explore whether higher levels of resilience are associated with the level of depression in breast cancer patients. The findings confirm the hypothesis that the level of resilience is negatively correlated with the level of depression in women treated for breast cancer. Higher levels of resilience may represent a protective factor from the emotional suffering of people who are dealing with significant stressful life event such as a cancer diagnosis. In our sample, the association between depression and resilience is statistically significant, although one can not reliably tell the direction of the connection between these two variables. On one hand it is possible that the low level of emotional distress causes higher levels of resilience, on the other hand the high level of resilience leads to less emotional suffering. Further longitudinal research is needed to examine whether high levels of resilience represent a long-term protection from depression/emotional suffering and to examine the relation of these two variables [34]. These findings should be evaluated with caution due to the relatively small sample and the cross-sectional design of the study; however they are a good basis for further research in this area.

In our sample, 12.4% of the patients suffered from major depression, and additional 14.7% suffered from depressive symptoms that altogether did not reach the criteria for major depression (subthreshold group). The levels of resilience are higher in not depressed group, than the subthreshold and major depression group. Also subthreshold depression group has better levels of resilience than major depression group. Our results show that even mild severity of depression reflects in the levels of resilience.

In our sample there was no statistical significance between the depression and time passed since diagnosis, and the stage of cancer p > 0.05. This finding is contrary to the literature data in which the level of depression decreases over time after the initial shock of the diagnosis and the treatment. More extensive research is required to reach a valid conclusion that will refer to our population.

If we collect more data from our population, about what helps oncology patients to be resilient, all healthcare professionals can create specific psychosocial interventions that target resilience. These interventions for enhancing psychological resilience may be a useful approach in overcoming the psychological distress in people who are dealing with breast cancer [35], to improve adherence to oncology treatments, and to improve the overall quality of life of the patients and their families.

In conclusion, this study shows that psychological resilience may independently contribute to lower levels of depression and psychological distress among patients treated for breast cancer. Patients who are less depressed have higher levels of resilience. More extensive research is required to verify the impact of higher levels of psychological distress on resilience and to establish the direction of the impact of these two phenomena. The level of psychological resilience may be a protective factor for depression and psychological distress.

Various psychosocial interventions to enhance psychological resilience may be useful approach in overcoming the psychological distress in people who are dealing with breast cancer. Based on data from our population, we can identify specific objectives for which psychosocial interventions can be implemented in clinical practice.
